# Characteristic Cardiac Magnetic Resonance (CMR) Imaging Findings of Cocaine-Induced Myocardial Injury

**DOI:** 10.7759/cureus.67072

**Published:** 2024-08-17

**Authors:** Jerry Fan, Laith Wahab, Vinh Nguyen

**Affiliations:** 1 Cardiology, Baylor Scott & White Medical Center - Temple, Temple, USA; 2 Internal Medicine, Baylor Scott & White Medical Center - Temple, Temple, USA

**Keywords:** cardiac magnetic resonance (cmr), cardiomyopathy, myocardial necrosis, myocardial injury, cocaine

## Abstract

Cocaine is a widely available illicit substance with a costly financial and social burden on the healthcare infrastructure. Both acute and chronic cocaine use can lead to sequelae of cardiac diseases, including myocardial infarction, aortic dissection, and cardiomyopathy. Cardiac magnetic resonance (CMR) imaging is a powerful tool for detecting myocardial injury leading to prompt treatment and risk stratification. We present two differing cases of sequelae of myocardial injury as a result of cocaine use. We present critical findings on CMR imaging, including myocardial injury patterns, which can help differentiate between acute and chronic injury and assess the extent of damage. Cocaine exerts potent sympathomimetic effects, increasing myocardial oxygen demand and causing coronary vasospasm, thrombosis, and direct myocyte toxicity. Acute cocaine use significantly elevates the risk of myocardial infarction, while chronic use can lead to cardiomyopathy and heart failure. CMR features include wall motion abnormalities, myocardial perfusion defects, and fibrosis. Early identification and intervention can potentially reverse interstitial fibrosis before progression to irreversible damage. CMR is an essential diagnostic tool for characterizing myocardial injury, distinguishing between reversible and irreversible damage, and providing prognostic information on cocaine-induced myocardial injury. The cases highlight the importance of CMR in managing and understanding the full spectrum of cocaine-related cardiac damage.

## Introduction

Cocaine is a popular and widely available illicit drug in the United States [1]. Approximately 6.4 million people in the United States use cocaine, accounting for 155-226 million dollars of annual healthcare expenditure for cocaine-related complications [1-2]. Cocaine-associated myocardial injury can be silent or manifest in overt complications, including hypertensive emergency, aortic dissection or rupture, malignant arrhythmias, cerebral hemorrhage, sudden cardiac death, myocarditis, myocardial infarction, cardiomyopathy, and endocarditis [3-4]. Approximately 71% of asymptomatic cocaine users have myocardial injuries, which can be divided into ischemic and non-ischemic consequences, manifesting as left ventricular dilation and dysfunction, hypertrophy, right ventricular dysfunction, and left ventricular edema and fibrosis [5]. Newer imaging modalities such as cardiovascular magnetic resonance imaging (CMR) can reliably detect early and late myocardial injury and distinguish between ischemic and non-ischemic injury [6]. We present two distinct cases of cocaine-induced myocardial injury that exemplify the spectrum of the disease.

## Case presentation

Case 1

**Figure 1:**
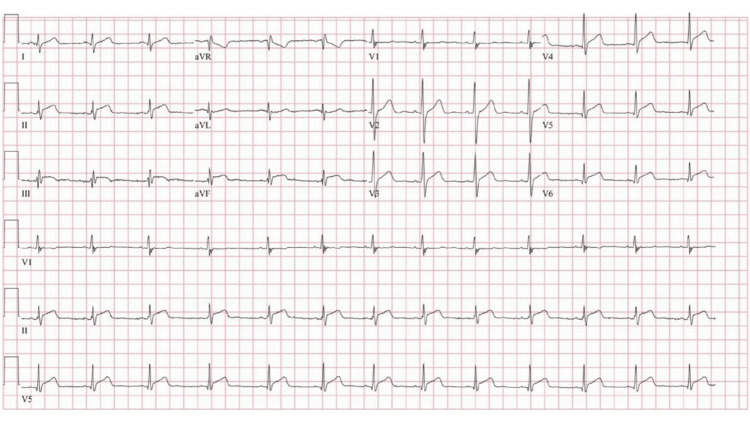
Electrocardiogram notable for diffuse ST-segment elevations anterolateral and inferior leads.

A 20-year-old man without a significant past medical history presented with substernal chest pain that worsened with deep inspiration and radiated to the left jaw and neck for the past six hours. Vital signs were normal. The urine drug screen was positive for cocaine and cannabinol. He acknowledged cocaine use, and his most recent use was two days prior. Peak troponin I was 52 ng/mL (normal 0.00-0.09 ng/mL). The electrocardiogram (ECG) showed diffuse ST-segment elevation (Figure 1).

**Figure 2:**
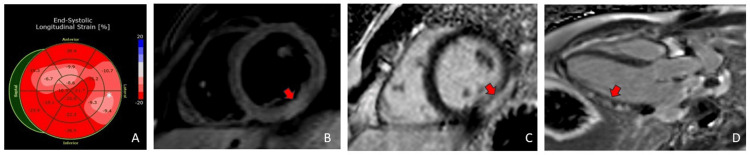
Panel A: Abnormal global longitudinal strain at the lateral wall. Panel B: Hypertense signal on T2-weighted imaging indicating the presence of myocardial edema (red arrow). Panel C: Increased native T1 and extracellular volume (ECV) indicating myocardial necrosis causing extracellular fraction expansion (red arrow). Panel D: Late gadolinium enhancement due to the delayed washout in the diseased myocardium; the injury pattern is most consistent with nonischemic based predominantly on the meso- and epicardial enhancement with relative sparing of the subendocardium (red arrow).

The transthoracic echocardiogram (TTE) demonstrated a normal left ventricular size and function with an ejection fraction of 55-60% and normal global longitudinal strain of -17.4% (normal -17% to -24% for males) but with diminished absolute values in the lateral wall (Figure 2). He underwent a coronary angiogram (CAG) for persistent chest pain that did not show angiographic evidence of coronary artery disease. He then underwent a CMR to evaluate for a non-ischemic cause of his myocardial injury. CMR demonstrated inferolateral hyperintensity on T2-weighted imaging consistent with edema. There was associated meso- and subepicardial gadolinium delayed enhancement at the same segment. Findings were most consistent with acute myocarditis (Figure 2).

Case 2

A 58-year-old man with longstanding polysubstance abuse that includes marijuana, cocaine, tobacco and alcohol use, and heart failure with reduced ejection fraction presented with substernal chest pain, dyspnea, peripheral edema, orthopnea, and paroxysmal nocturnal dyspnea. The last cocaine use was two days prior. Vital signs were normal. The ECG demonstrated left ventricular hypertrophy and non-specific ST-T wave abnormalities. Troponin I and brain natriuretic peptide levels were normal.

He was initially diagnosed with heart failure with reduced ejection fraction (LVEF 25%) six years ago after an acute episode of chest pain following cocaine use. Unfortunately, socioeconomic factors did not permit medication adherence, and he was transiently homeless. A transthoracic echocardiogram during the current admission showed an interval improvement in LVEF to 40-45% (during a subsequent admission for heart failure) despite ongoing use of cocaine. A CAG was negative for an obstructive disease. He underwent CMR to further characterize heart failure.

**Figure 3:**
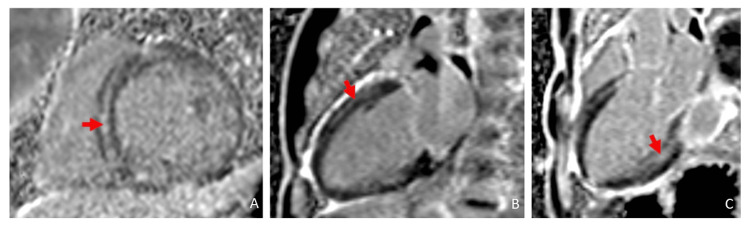
Cardiac magnetic resonance with multifocal late gadolinium enhancement noted at the septal mesocardium (red arrow, Panel A), anterior mesocardium (red arrow, Panel B), and inferolateral subendocardium (red arrow, Panel C).

CMR demonstrated a mildly dilated left ventricle with global hypokinesis (LVEF 34%). There was markedly abnormal multifocal late gadolinium enhancement involving multiple myocardial layers (Figure 3).

**Figure 4:**
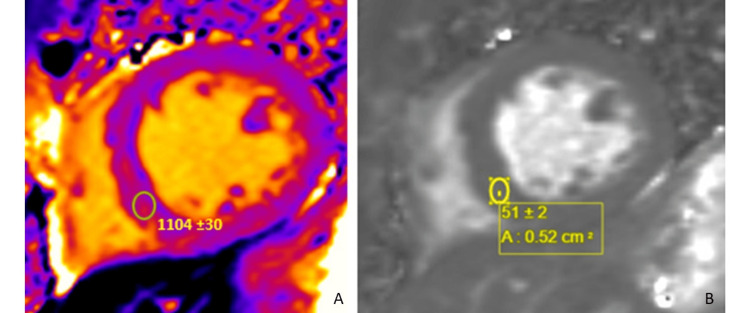
Panel A: Elevated native T1 mapping (normal <1050 ms) indicative of increased interstitial expansion, with an extracellular volume (ECV) fraction of 47%. Panel B: T2 mapping noted to be normal (normal <64 ms), excluding acute edema/inflammation.

The findings were most consistent with substance abuse associated with catecholaminergic myocardial injury (mid-wall late gadolinium enhancement (LGE)) and vasospastic infarction (subendocardial late gadolinium enhancement). There was expanded interstitial volume (ECV of 47%) quantified at the septal segment, which indicated replacement fibrosis (Figure 4). There was no evidence of acute edema/inflammation based on a normal T2 mapping. He was discharged on guideline-directed medical therapy and substance abuse counseling and rehabilitation. He subsequently was admitted to a halfway house for substance abuse rehabilitation.

## Discussion

Cocaine is a potent sympathomimetic agent, which inhibits the reuptake of norepinephrine and dopamine in the presynaptic terminal resulting in the accumulation of catecholamines in the post-synaptic terminal [3,7]. The sympathomimetic effect of cocaine increases the heart rate and blood pressure, which increases myocardial oxygen demand [3,7]. The accumulation of potent vasoconstrictors in the post-synaptic terminal causes platelet adherence and increases thrombus formation and coronary vasospasm [3,7]. This leads to a decrease in oxygen supply to the myocardium, resulting in ischemia and infarction [3,7]. Cocaine also has direct cytotoxic effects on myocytes through impaired nitric oxide release [2-3]. Cocaine-induced ischemic and nonischemic insults can be reliably assessed by CMR [2,6]. The acute and chronic effects of cocaine on the myocardium and blood vessels are summarized below (Figure 5).

**Table 1:** Effects of cocaine on the heart and blood vessels

Acute	Chronic
↑ Heart rate	Dilated cardiomyopathy due to the long-term myocardial oxygen mismatch
↑ Blood pressure	Left ventricular hypertrophy
↑ Myocardial contractility	Myocarditis
↑ Coronary spasm / vasoconstriction	Accelerated atherosclerosis
↑ Platelet aggregation / thrombosis	

The CMR features of cocaine-induced myocardial injury include wall motion abnormalities, pericardial effusion, reduced myocardial perfusion, inflammation, and disease processes involving coronary arteries [3,6]. CMR sequences to assess myocardial injury include cine steady state-free precession frequency (ssfp), myocardial perfusion, T2-weighted imaging, T1-weighted imaging such as T1 mapping, and LGE [6]. Cine steady-state free precession (SSFP) MRI is a type of gradient-echo MRI pulse sequence in which a steady, residual transverse magnetization (Mxy) is maintained between successive cycles [6]. The sequence is noted for its superiority in the dynamic/cine assessment of cardiac function [6]. Myocardial perfusion imaging allows live visualization, of the lack of contrast uptake by the myocardial segments [6]. T2-weighted sequences such as triple inversion recovery and T2 mapping reflect the water composition of the myocardium and are a marker of acuity [6]. It is used to assess myocardial edema/inflammation in acute pathologies such as ischemic and nonischemic toxic myocyte damage [3]. Myocardial injury patterns with normal T2-weighted imaging suggest a nonacute process [6]. T1 mapping allows the differentiation of various tissues (fat, myocardium, and fluid) based on the tissue-specific T1 relaxation time [6]. T1 mapping can also be used to quantify the ECV fraction of the myocardium [6]. Interstitial fibrosis, considered reversible fibrosis, is the hallmark of all myopathic processes and is a major contributor to the ECV [6]. Identification of an expanded ECV gives an opportunity to halt the disease process and potentially reverse interstitial fibrosis before progression to myocyte death or replacement fibrosis [6]. LGE imaging is obtained around 10 minutes after gadolinium contrast injection. Contrast should have washed out by 10 minutes, but regions of replacement fibrosis with collagen abundance sequester contrast and remain enhanced. The pattern of LGE reflects the mechanism of injury. A subendocardial LGE indicates an ischemic injury, whereas mid-wall or subepicardial LGE indicates a nonischemic injury (myocarditis or direct myocardial injury by an offending agent) [6]. LGE can be due to an acute injury (necrosis) or remote injury (fibrosis) [6]. T2-weighted imaging for edema is interpreted in the constellation of LGE to determine the acuity of the disease [6].

The acute phase of cocaine use increases the incidence of myocardial infarction by 24-fold in the first hour after use but quickly drops off to fourfold risk by the second hour [4]. The proposed mechanism for myocardial infarction involves coronary thrombosis due to inflammatory changes in the media and intima rather than plaque rupture [4]. This manifests in similar subendocardial or transmural enhancement in a coronary distribution detectable by CMR [4]. Ischemic changes caused by intense vasoconstriction can be detected on first-pass perfusion imaging as areas of perfusion defect. Regional wall motion abnormalities can be assessed using cine imaging, which allows the visualization of hypokinetic or akinetic regions [2-3].

Non-ischemic changes to the myocardium usually result from long-term chronic use of cocaine resulting in cardiomyopathy [2-3]. Reduction in systolic and diastolic function, left and right ventricular hypertrophy, and dilation lead to chronic heart failure [3]. Long-term exposure to catecholamines results in degenerative and inflammatory alterations in the myocardium [3]. Upwards of 71% of asymptomatic cocaine users will have CMR changes associated with myocardial edema and fibrosis despite remaining clinically asymptomatic [3,6-7].

Cocaine-induced myocarditis has characteristic findings on CMR [6]. CMR distinguishes early and late consequences of myocardial inflammation and is a Class I recommendation for the evaluation of myocarditis [6]. Early myocardial injury is the result of inflammatory injury causing interstitial edema and myocyte necrosis, which later evolve into late findings of fibrosis leading to left ventricular systolic dysfunction [6]. In cocaine-induced myocardial injury, LGE may demonstrate focal enhancement within the myocardium, representing areas of fibrosis and necrosis [6]. This enhancement pattern may be subendocardial, transmural, or patchy depending on the extent and severity of the injury [6]. A repeat CMR in four to eight months can be useful for determining prognosis based on persistence versus the resolution of myocardial injury [3].

## Conclusions

The utilization of CMR serves as a crucial diagnostic tool to characterize the myocardial tissue, offering insights into the spectrum of myocardial damage. Cocaine-induced myocardial injury is often asymptomatic with only a minority of patients presenting with overt myocardial damage in the form of myocarditis, myocardial ischemia, or infarction. CMR not only aids in distinguishing reversible from irreversible damage but also provides valuable prognostic information. Furthermore, it was suggested to repeat CMR after four to eight months of appropriate management to evaluate myocardial response to abstinence and medical therapy.
